# Cardiovascular Disease from Pathophysiology to Risk Estimation: Is Inflammation Estimated through Perivascular Attenuation on Computed Tomography the Key?

**DOI:** 10.3390/life14040457

**Published:** 2024-03-29

**Authors:** Domenico Tuttolomondo, Giampaolo Niccoli, Chiara Martini, Fabrizio D’Ascenzo, Ovidio De Filippo, Francesco Nicolini, Francesco Formica, Davide Carino, Filippo Luca Gurgoglione, Andrea Denegri, Giulia Magnani, Luigi Vignali, Massimo De Filippo, Nicola Sverzellati, Andrea Ticinesi, Luca Bergamaschi, Carmine Pizzi, Elisa Gherbesi, Sergio Suma, Nicola Gaibazzi

**Affiliations:** 1 Department of Cardiology, Parma University Hospital, Via Gramsci 14, 43126 Parma, Italy; 2 Department of Diagnostic, Parma University Hospital, 43126 Parma, Italy; 3 Department of Medicine and Surgery, University of Parma, Via Antonio Gramsci 14, 43126 Parma, Italy; 4 Division of Cardiology, Cardiovascular and Thoracic Department, Città della Salute e della Scienza, 10126 Turin, Italy; 5 Department of Cardiac Surgery, Parma University Hospital, Via Gramsci 14, 43126 Parma, Italy; 6 Department of Medicine and Surgery (DiMec), Section of Radiology, University of Parma, Maggiore Hospital, Via Gramsci 14, 43126 Parma, Italy; 7 Scienze Radiologiche, Dipartimento di Medicina e Chirurgia, University-Hospital of Parma, 43126 Parma, Italy; 8 Geriatric-Rehabilitation Department, Azienda Ospedaliero-Universitaria di Parma, 43126 Parma, Italy; 9 Cardiology Unit, IRCCS Azienda Ospedaliera-Universitaria di Bologna, 40138 Bologna, Italy; 10 Department of Medical and Surgical Sciences—DIMEC—Alma Mater Studiorum, University of Bologna, 40138 Bologna, Italy; 11 Department of Cardio-Thoracic-Vascular Diseases, Foundation IRCCS Ca’ Granda Ospedale Maggiore Policlinico, 20154 Milan, Italy

**Keywords:** perivascular adipose tissue attenuation, pericoronary adipose tissue attenuation, computed tomography, all-cause mortality, cardiovascular mortality, atrial fibrillation, aortic aneurysm, atherosclerotic plaques, coronary microvascular dysfunction

## Abstract

(1) Background: Systemic inflammation stands as a well-established risk factor for ischemic cardiovascular disease, as well as a contributing factor in the development of cardiac arrhythmias, notably atrial fibrillation. Furthermore, scientific studies have brought to light the pivotal role of localized vascular inflammation in the initiation, progression, and destabilization of coronary atherosclerotic disease. (2) Methods: We comprehensively review recent, yet robust, scientific evidence elucidating the use of perivascular adipose tissue attenuation measurement on computed tomography applied to key anatomical sites. Specifically, the investigation extends to the internal carotid artery, aorta, left atrium, and coronary arteries. (3) Conclusions: The examination of perivascular adipose tissue attenuation emerges as a non-invasive and indirect means of estimating localized perivascular inflammation. This measure is quantified in Hounsfield units, indicative of the inflammatory response elicited by dense adipose tissue near the vessel or the atrium. Particularly noteworthy is its potential utility in assessing inflammatory processes within the coronary arteries, evaluating coronary microvascular dysfunction, appraising conditions within the aorta and carotid arteries, and discerning inflammatory states within the atria, especially in patients with atrial fibrillation. The widespread applicability of perivascular adipose tissue attenuation measurement underscores its significance as a diagnostic tool with considerable potential for enhancing our understanding and management of cardiovascular diseases.

## 1. Introduction

Systemic inflammation is a known independent cardiovascular risk factor for atherosclerotic disease that plays an important role in the determination of most major cardiovascular events. This influence is likely exerted through increased inflammation in atherosclerotic plaques.

Most known cardiovascular risk factors are in fact associated with increased inflammation, as follows:

Tobacco habit leads to airway inflammation and predisposes to lung diseases such as obstructive pulmonary disease, which is also a risk factor for ischemic cardiovascular disease, where there is a dysregulation of the immune system and drugs with immunomodulatory action are often among the treatments [1,2].

Social deprivation, that is, lack of access to essential social and economic resources, creates an environment that fosters chronic stress; excessive stress triggers a cascade of systemic inflammatory response, leading to an upregulation of inflammatory markers [3].

Obstructive sleep apnea syndrome favors the increased secretion of catecholaminergic hormones that tend to increase systemic blood pressure values and promote systemic inflammation, thus leading to a dual risk [4,5].

The abundance of refined foods, typically high in processed sugars and low in essential nutrients, contributes significantly to an overall increase in caloric intake; this dietary pattern, marked by an excessive intake of calories and saturated fats, synergizes with a sedentary lifestyle to promote weight gain, obesity, and increased inflammatory status [6].

The increase in the percentage of adipose tissue leads to a greater secretion of hormones such as adiponectin which on the one hand increases the extent of the systemic inflammatory response [7,8].

Patients with diabetes mellitus exhibit a dysregulated immune system, with the pathogenetic and proinflammatory influence of glycated hemoglobin playing a pivotal role in both the initiation and subsequent expansion of atherosclerotic plaque formation [9].

One of the most important cardiovascular risks factors is the increased level of low-density lipoproteins (LDL); a high concentration of LDL in the coronary atherosclerotic plaque triggers a localized inflammatory response driven by macrophages which implements the progression of the plaque itself [10,11]. Lipoprotein(a) is also known to act through vascular inflammation [12].

Patients with autoimmune diseases characterized by persistent elevation in inflammation levels are at increased susceptibility to develop ischemic heart disease. It is postulated that the chronic upregulation of inflammation not only causes the primary autoimmune condition but also creates a scenario that facilitates the risk of ischemic events, establishing a complex interplay between immune dysregulation and cardiovascular health [13].

The convergence of diverse risk factors appears to contribute to a common pathway, ultimately amplifying the inflammatory response.

In this scenario, the potential dismal role of localized vascular inflammation in the genesis and promotion of cardiovascular disease appears to be of primary importance and should be re-evaluated now that the examination of perivascular adipose tissue attenuation (PVAT) on computed tomography (CT) scans emerges as a non-invasive, although indirect, means of estimating localized perivascular inflammation.

## 2. Discussion

### 2.1. Is Inflammation Truly Involved in Ischemic Heart Disease?

The CANTOS trial showed that therapy with Canakinumab, a therapeutic monoclonal antibody targeting interleukin-1β, administered in patients with an earlier myocardial infarction and C-reactive protein (CRP) values of at least 2 mg/L reduced the incidence of the composite endpoint of non-fatal myocardial infarction, non-fatal stroke, and death from cardiovascular causes, but increased the risk of fatal infections [14]. In the Colchicine Cardiovascular Outcomes (COLCOT) trial, which enrolled 4745 patients with acute myocardial infarction in the previous 30 days, low-dose Colchicine (0.5 mg daily) was associated with a significant reduction in the primary composite endpoint (cardiovascular death, resuscitated cardiac arrest, MI, stroke, or urgent revascularization) in comparison to placebo [15]. Similarly, the LoDoCo2 trial shows that low-dose Colchicine administered in patients with known chronic coronary syndrome reduces the composite endpoint of cardiovascular death, spontaneous myocardial infarction, ischemic stroke, or ischemia-driven coronary revascularization compared to placebo; there was also a significant reduction in the secondary composite endpoint (the same as the primary composite endpoint of the CANTOS trial) with a trend towards higher non-cardiovascular mortality in the colchicine-treated group (hazard ratio, 1.51; 95% CI, 0.99 to 2.31) [16].

Based on these findings, the recent European Society of Cardiology (ESC) guidelines suggest that therapy with Colchicine may be considered in long-term management of acute coronary syndrome, especially when other risk factors are insufficiently controlled, or recurrent cardiovascular disease events occur on optimal medical therapy; this is the first time that ESC guidelines indicate immunomodulating therapy for the treatment of ischemic heart disease [17].

The role of inflammation acts through multiple pathways that can potentially have an additive effect promoting ischemic cardiovascular disease, and the link between inflammation and myocardial infarction is also somehow indirectly proven by the effectiveness of immunomodulating drugs in secondary prevention.

### 2.2. What Is Perivascular Adipose Tissue Attenuation on Computed Tomography and Why Is It Supposed to Measure Inflammation?

Perivascular adipose tissue, once relegated to the status of an inert anatomical feature, has evolved into a recognized and dynamic component with far-reaching physiological implications. This specialized fat accumulation, positioned close in contact to blood vessels, is now acknowledged as a paracrine organ, contributing significantly to the intricate regulation of vascular biology. Its transformative characterization stems from the realization that perivascular fat is not merely a passive bystander but an active participant, orchestrating the secretion of a diverse array of biologically active molecules. The intricate interplay of molecular signals emanating from perivascular adipose tissue has broad implications for vascular health and disease, marking a departure from the traditional perception of adipose tissue as a static repository of energy [18]. Examining the attenuation of PVAT has emerged as a non-invasive and indirect method for estimating localized perivascular inflammation. This assessment is quantified in Hounsfield units, reflecting the inflammatory response triggered by dense adipose tissue near the vessel or atrium.

PVAT estimation is made possible through post-processing of CT scans, showcasing the evolving capabilities of imaging technologies in capturing and quantifying subtle yet clinically relevant phenomena. Notably, the measurement of PVAT on CT scans demonstrates good inter-observer reproducibility, enhancing its credibility as a reliable and standardized tool in the assessment of vascular inflammation [19].

PVAT measurement on CT scans not only provides a window into the dynamic nature of perivascular adipose tissue but also holds promise as a valuable diagnostic and prognostic marker.

Table 1 shows all the characteristics and studies included in the current review in which PVAT was measured.

### 2.3. Perivascular Adipose Tissue Attenuation on Internal Carotid Artery

In patients with ischemic stroke or transient ischemic attack not from cardioembolic cause, computed tomography angiography of the carotid arteries is performed routinely.

A higher PVAT on stenotic internal carotid artery was associated with the presence of cerebrovascular symptoms in patients with transient ischemic attack or non-cardioembolic ischemic stroke. Furthermore, the PVAT applied on the internal carotid artery with spontaneous dissection is greater than on the contralateral vessel in the same subject [20,21,22].

### 2.4. Perivascular Adipose Tissue Attenuation on Aorta

Rodríguez-Granillo and collaborators examined the density of periaortic adipose tissue on CT in patients suffering from cardioembolic, non-cardioembolic, and unclear genesis strokes showing that the aortic PVAT is greater in patients with cardioembolic stroke [23].

**Figure 1:**
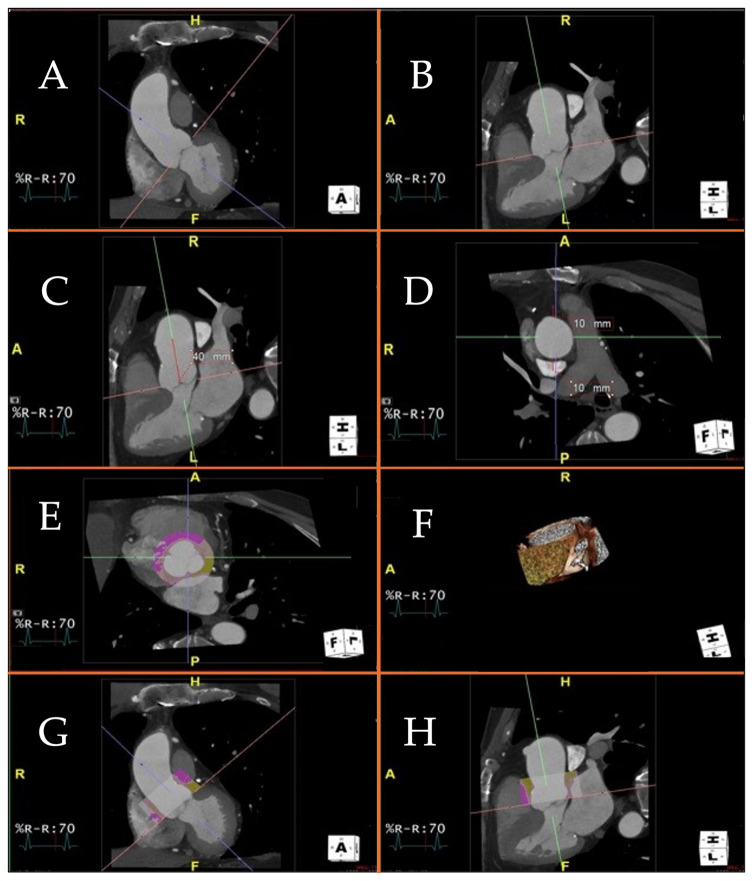
Figure 1 displays a visualization of the cardiac planes and ascending aorta using a 3D platform and the MultiPlanar Reconstruction (MPR) application. Specifically, the MPR mode enables accurate positioning of the cardiac viewing planes for the calculation of perivascular adipose tissue attenuation. Technical steps required to measure perivascular adipose tissue attenuation of the proximal ascending thoracic aorta on chest computed tomography angiography. (**A**–**C**): different projections of the ascending aorta and aortic valve. You can see the projections in two (**A**) and three (**B**,**C**) chambers, respectively; (**D**): axial visualization of the ascending aorta, at the pulmonary trunk; (**E**): Axial visualization of the aortic valve, at the three cusps, in diastolic phase. The colored circular section indicates the application of the software to measure perivascular adipose tissue attenuation of the proximal ascending thoracic aorta and aortic bulb; (**F**): Visualization volume rendering of the aortic bulb, at the origin of the coronary arteries; (**G**): Two-chamber visualization of the ascending aorta. Colored section indicates software application to measure perivascular adipose tissue attenuation of the proximal ascending thoracic aorta and aortic bulb; (**H**): Three-chamber visualization of the ascending aorta. Colored section indicates software application to measure perivascular adipose tissue attenuation of the proximal ascending thoracic aorta and aortic bulb.

Patients with an aneurysm of the ascending thoracic aorta (AAA) show significantly elevated PVAT values compared to controls, and histopathological analysis of AAA patients undergoing surgery shows a correlation between increased PVAT and the degree of fibrosis of the vascular wall [24,31]. In view of the long-time course required for the genesis of AAA, it is hypothesized that vascular fibrosis is the late product of a prior inflammatory process. Figure 1 shows the process used to estimate the perivascular attenuation in the first 40 mm of the ascending thoracic aorta by Gaibazzi and collaborators [24,31].

Figure 1A shows proper alignment of the planes in the aortic valve plane. In Figure 1B, the proximal ascending thoracic aorta is selected for a length of 40 mm from the aortic valve plane. Figure 1C shows the short axis of the ascending thoracic aorta, i.e., seen through a horizontal computed tomography plane; a region of interest 10 mm radial to the vessel is then selected. Figure 1D shows the aortic perivascular tissue at the level of the aortic valve plane, the sagittal plane, and the coronal plane; in yellow is the perivascular tissue with a density between −30 and −190 Hounsfield units, which corresponds to adipose tissue. In the upper right 1D box, the 3D reconstruction of the region of interest is shown, i.e., the perivascular adipose tissue of the first 40 mm of the proximal ascending thoracic aorta.

Yamaguchi and his team saw a correlation between an increase in aortic PVAT and progression of the aneurysm in a small group of patients (n = 77) with abdominal aortic aneurysm (AbAA) [25].

Dias-Neto and collaborators compared the PVAT in diseased and non-diseased aortic segments within the same individuals with nontreated asymptomatic AbAA, aorto-iliac occlusive disease, and individuals without evidence of aortic disease showing a significantly higher intra-individual PVAT difference in patients with AbAA rather than in the other two groups [26].

### 2.5. Inflammation and Atrial Fibrillation

The comprehensive guidelines on the diagnosis and management of atrial fibrillation, as delineated by the European Society of Cardiology in 2020, underscore the intricate and multifactorial nature of this prevalent cardiac arrhythmia. Beyond a single etiological factor, these guidelines illuminate a confluence of diverse contributors to the onset and progression of atrial fibrillation. Among the array of risk factors implicated in this complex interplay, inflammatory diseases assume a prominent role, standing alongside other notable contributors such as tobacco habit, obstructive pulmonary disease, obstructive sleep apnea syndrome, obesity, diabetes mellitus, and dyslipidemia [32].

The recognition of inflammatory diseases as one facet of the multifactorial genesis of atrial fibrillation aligns with an evolving understanding of the interconnected nature of cardiovascular health and systemic inflammation. The acknowledgment of the role of inflammatory diseases in this context highlights the need for a holistic approach in both diagnosis and management. Furthermore, the interwoven nature of tobacco habit, obstructive pulmonary disease, obstructive sleep apnea syndrome, obesity, diabetes mellitus, dyslipidemia, and inflammatory diseases is exemplified by their shared characteristic of fostering an increased systemic inflammatory state [1,2,4,5,6,7,9,10,11,12,13].

It has been shown on large general population cohorts of 47,000 individuals that systemic inflammation, estimated by CRP, increases the risk of developing atrial fibrillation independently of other risk factors, but not in subjects with elevated CRP levels due to genetic causes [33]. In a cohort of 2434 women, a significant relation between inflammatory markers such as CRP, fibrinogen, and soluble intercellular adhesion molecule-1 and new onset atrial fibrillation at a median follow-up of 14.4 years was proven [34].

Considering the well-established association between systemic inflammation and the onset of atrial fibrillation, a logical extension of this understanding prompts consideration of the potential impact of inflammation localized specifically around the atria. This localized inflammatory state is postulated to exert a substantive influence on both the initiation and sustained maintenance of atrial fibrillation. As the scientific community delves deeper into the complexities of atrial fibrillation pathophysiology, it becomes increasingly apparent that understanding the interplay between systemic and localized inflammation is pivotal in comprehending the multifactorial nature of this cardiac arrhythmia.

Indeed, peri-atrial inflammation measured non-invasively through attenuation of left retro-atrial adipose tissue on myocardial CT is associated with atrial fibrillation, independently of left atrial size [27,35].

This finding not only enriches our understanding of atrial fibrillation pathophysiology but also holds promise for the development of tailored interventions potentially orienting more effective strategies for the prevention and management of atrial fibrillation.

### 2.6. Pericoronary Adipose Tissue Attenuation

In patients with suspected chronic coronary syndrome, different diagnostic tests can be used, such as stress echocardiogram and coronary CT. The appropriate selection of the diagnostic method should be tailored to the patient’s characteristics, method availability, and the expertise of the local center [36,37,38,39,40]. Coronary CT is a valid and widely used method in daily clinical practice; it allows the evaluation of the presence of significant coronary stenoses as well as high-risk plaques characterized by positive remodeling, low Hounsfield units, napkin-ring sign, and the presence of intra-plaque spotty calcifications [41,42,43].

The correlation between local inflammation at histopathologic assessment in pericoronary adipose tissue and the degree of PCAT is now proven [44]. The role of pericoronary adipose tissue attenuation (PCAT) in terms of prognostic stratification has been widely proven on top of other prognostic signs on coronary CT, for both cardiovascular and all-cause mortality [28]. PCAT was measured on all coronary arteries, but it stratified cardiovascular and all-cause mortality only when tested on the left anterior descending artery (LAD) and right coronary artery; in fact, PCAT can be used indifferently on either the LAD or the right coronary artery. PCAT measured on the circumflex artery stratifies all-cause but not cardiovascular mortality, which is why the PCAT estimate on the circumflex artery as of today should probably avoided until not proven otherwise. The method for PCAT measurement was originally described by Oikonomu et al. [28].

Although Canakinumab has been shown to reduce the risk of recurrence of acute myocardial infarction, this therapy is not currently indicated in the ESC guidelines because it may increase the risk of fatal cancer, fatal infections, and thrombocytopenia.

It should be considered that patients in the CANTOS trial were not selected based on the presence of localized coronary inflammation, as was also carried out in the COLCOT trial [14,15,16].

Potentially initiating immunomodulatory therapy in the context of chronic coronary syndrome based on the presence of coronary inflammation estimated by PCAT could be the basis for personalizing anti-inflammatory therapy only in those patients who can benefit most.

This approach is feasible today and we speculate it would potentially help to use such drugs only when useful, perhaps favorably increasing their therapeutic/adverse effects balance.

### 2.7. PeriCoronary Adipose Tissue Attenuation in COVID-19

COVID-19 is also a systemic inflammatory disease that in addition to pulmonary involvement in the most severe forms predisposes to cardiovascular events such as myocardial damage and vascular thrombosis [45,46,47]. The underlying mechanism of myocardial damage is still unclear, although it can be hypothesized that it is either due to direct damage from virus localization in myocardial cells or to hypoxia resulting from impaired lung function, but the prevailing hypothesis is the destabilization of pre-existing atherosclerotic coronary plaques favored by extreme inflammation [48].

In hospitalized COVID-19 patients, the most impactful treatment involves the use of immunosuppressive and antiviral medications rather than antibiotics. This therapeutic approach has shown significant improvements in patient outcomes [49,50,51,52,53].

A recent study aimed to elucidate the potential prognostic role of PCAT in COVID-19 patients requiring hospitalization. PCAT appeared to be independently associated with higher mortality in patients with severe COVID-19, while the pre-existent coronary atherosclerotic burden, measured as Agatston calcium score, was instead not associated with adverse outcomes after adjustment for covariates [29].

### 2.8. Coronary Microvascular Dysfunction and Inflammation

Microvascular dysfunction and chronic inflammation represent pervasive and central disease mechanisms, exerting a pivotal influence in many cardiovascular conditions, ranging from coronary artery disease to heart failure [54,55]. Furthermore, they are involved in a diverse array of non-cardiovascular diseases, ranging from cancer to dementia [56,57]. The human microcirculation, a complex network of very small vessels and capillaries, plays a crucial role in tissue perfusion and is also recognized as a paracrine modulator of the local tissue environment. This modulation is instrumental in the initiation and progression of several chronic and life-threatening diseases, highlighting its importance as a therapeutic target. Interestingly, microcirculatory dysfunction is intrinsically associated with chronic inflammation, which serves as a key pathogenic factor shared across the entire spectrum of diverse diseases mentioned. As such, the functional status of the microcirculation may serve as a valuable indicator of the patient’s inflammatory state, offering potential insights into their overall health and prognosis [58,59].

The development of abnormal vasomotion can be attributed to an intricate and bidirectional interplay between inflammation and oxidative stress. Within this dynamic interaction, elevated levels of reactive oxygen species in coronary arterioles can detrimentally impact vascular function. This occurs through a multifaceted process involving the reduction in the production or availability of nitric oxide, ultimately leading to the impairment of both endothelium-dependent and endothelium-independent nitric oxide-mediated vasodilation. In essence, the interrelationship between inflammation and oxidative stress sets the stage for the disruption of normal vasomotion, with reactive oxygen species acting as a critical mediator in the functional decline of the vascular endothelium [60].

Coronary flow velocity reserve measured in the left anterior descending artery during stress echocardiography with Dipyridamole infused at the total dose of 0.84 mg/kg is a non-invasive parameter to assess epicardial and microcirculatory coronary function [61,62].

Indeed, existing evidence has established that a reduced coronary flow velocity reserve in the left anterior descending artery, as identified in stress echocardiography, serves as a predictor of mortality across a spectrum of health conditions, encompassing cardiovascular causes, cancer, and all-cause mortality [63,64,65]. This supports the hypothesis that inflammation may be the common denominator driving coronary microcirculatory dysfunction in a multitude of pathological scenarios.

Hence, it can be postulated that inflammation plays a central role in the initiation and perpetuation of microvascular dysfunction across various disease states, thereby contributing to the increased mortality risk, either cardiovascular, cancer-related, and all-cause mortality. Digging deeper into the potential relationship between inflammation and microcirculatory dysfunction, we may unveil shared mechanistic pathways that may shed light on outcomes within a wide range of medical challenges.

Furthermore, it has been shown that PCAT correlates with microcirculatory dysfunction both in patients with and without obstructive-grade coronary atherosclerotic disease [30,66]. In fact, in patients with microvascular angina, coronary microvascular dysfunction may also be influenced by inflammatory changes that are potentially reflected in the increased inflammation of coronary vessels detected by PCAT.

## 3. Conclusions

The intricate interplay between systemic and localized vascular inflammation stands as a pivotal determinant in the complex etiology of cardiovascular disease, coronary microcirculatory dysfunction, and atrial fibrillation. This nuanced relationship underscores the need for advanced diagnostic tools that can illuminate the inflammatory landscape within the vasculature. In this context, the attenuation of pericoronary adipose tissue on CT emerges as a non-invasive method of paramount significance, offering an indirect method for estimating the extent of vascular inflammation, with a concurrent robust prognostic role. The application of PCAT on CT provides valuable insights into the inflammatory status of coronary arteries. Importantly, the identified prognostic role adds an additional layer of clinical utility, enabling healthcare practitioners to anticipate and manage cardiovascular risks more effectively. While its prognostic significance is well established within the coronary arteries, the extrapolation of its predictive power to other vascular districts remains an area where comprehensive investigation is imperative. Consequently, additional studies are warranted to elucidate the prognostic role of perivascular attenuation on CT in diverse vascular territories beyond the coronary arteries. In summary, the attenuation of pericoronary adipose tissue on CT stands as a promising frontier in cardiovascular research, providing a non-invasive window into vascular inflammation with notable prognostic implications. While its efficacy is firmly established in coronary arteries, ongoing research is crucial to unveil its potential prognostic utility in other vascular districts, thereby advancing our capabilities in predicting, preventing, and managing cardiovascular diseases more comprehensively.

## Figures and Tables

**Table 1:** General characteristics of selected studies and the vascular districts where the perivascular attenuation measurement on CT was performed.

Reference	Title	Authors	Year of Publication	Subjects	Vascular District	Results and Statistical Significance
[20]	Association Between Carotid Artery Perivascular Fat Density and Cerebrovascular Ischemic Events.	Baradaran et al.	2018	94 subjects	Internal carotid artery	In the between-subject analysis of stenotic ICAs, symptomatic patients had higher mean pericarotid fat density compared with asymptomatic patients (−66.2 ± 19.2 HU vs. −77.1 ± 20.4 HU, *p* = 0.009).
[21]	Identification of high-risk carotid plaque by using carotid perivascular fat density on computed tomography angiography.	Zhang et al.	2022	206 subjects	Carotid artery	Plaques with symptoms (−55.0 ± 10.0 HU) had a higher pericarotid fat density than those without symptoms (−68.0 ± 10.3 HU, *p* < 0.001).
[22]	Association between spontaneous internal carotid artery dissection and perivascular adipose tissue attenuation on computed tomography angiography.	Cheng et al.	2023	56 subjects	Internal carotid artery	Pericarotid fat density was significantly higher around dissected ICA compared with non-dissected contralateral ICA in the same patients (−58.7 ± 10.2 vs. −68.9 ± 8.1 HU, *p* < 0.0001) and ICA of patients without dissection (−58.7 ± 10.2 vs. −69.3 ± 9.3 HU, *p* < 0.0001).
[23]	Epicardial and periaortic fat characteristics in ischemic stroke: Relationship with stroke etiology and calcification burden.	Rodríguez-Granillo et al.	2022	182 subjects	Thoracic aorta	Patients with cardioembolic stroke had higher periaortic adipose tissue attenuation (non-CE −84.4 ± 7.0 HU vs. CE −78.1 ± 9.9 HU vs. ESUS −82.3 ± 9.3 HU, *p* < 0.001).
[24]	Attenuation of peri-vascular fat at computed tomography to measure inflammation in ascending aorta aneurysms.	Gaibazzi et al.	2021	240 subjects	Ascending thoracic aorta	Patients with ascending aorta aneurysm had higher periaortic adipose tissue attenuation compared with normal diameter of the ascending aorta (−69.1 HU vs. −75.1 HU *p* < 0.0001).
[25]	Clinical significance of increased computed tomography attenuation of periaortic adipose tissue in patients with abdominal aortic aneurysms.	Yamaguchi et al.	2021	77 subjects	Abdominal aorta	Periaortic adipose tissue attenuation is an independent predictor of abdominal aortic aneurysm progression (OR 1.12; 95% CI 1.05–1.20; *p* = 0.004).
[26]	High Density of Periaortic Adipose Tissue in Abdominal Aortic Aneurysm.	Dias-Neto et al.	2018	341 subjects	Abdominal aorta	Patients with abdominal aortic aneurysm presented higher intra-individual periaortic adipose tissue attenuation differences, with higher periaortic adipose tissue attenuation density around the aneurysm sac than the healthy neck.
[27]	Atrial Fibrillation and Peri-Atrial Inflammation Measured through Adipose Tissue Attenuation on Cardiac Computed Tomography.	Gaibazzi et al.	2021	160 subjects	Left atrium	Mean adipose tissue located around the left atrium density was significantly higher in AF (−69.15 HU) versus non-AF (−76.82 HU, *p* < 0.0001) participants.
[28]	Non-invasive detection of coronary inflammation using computed tomography and prediction of residual cardiovascular risk (the CRISP CT study): a post-hoc analysis of prospective outcome data.	Antonopoulos et al.	2017	3912 subjects	All coronary artery	PCAT measured around the right coronary artery predicts cardiac mortality, HR 2.15, 95% CI 1.33–3.48; *p* = 0.0017 in the derivation cohort, and HR 2.06, 1.50–2.83; *p* < 0.0001 in the validation cohort.
[29]	Coronary inflammation on chest computed tomography and COVID-19 mortality.	Tuttolomondo et al.	2024	769 subjects	Left anterior descending coronary artery	PCAT, but not the pre-existent coronary atherosclerotic burden, was independently associated with in-hospital mortality (*p* < 0.001).
[30]	Human coronary inflammation by computed tomography: Relationship with coronary microvascular dysfunction.	Pasqualetto et al.	2021	202 subjects	Left anterior descending coronary artery	The relationship between PCAT and CFVR-lad show a significant inverse relationship in the entire group of subjects enrolled in the study (r = −0.32, *p* < 0.001).
